# Guidelines for Patient-Centered Documentation in the Era of Open Notes: Qualitative Study

**DOI:** 10.2196/59301

**Published:** 2025-01-20

**Authors:** Anita Vanka, Katherine T Johnston, Tom Delbanco, Catherine M DesRoches, Annalays Garcia, Liz Salmi, Charlotte Blease

**Affiliations:** 1 Division of General Medicine Department of Medicine Beth Israel Deaconess Medical Center Boston, MA United States; 2 Harvard Medical School Boston, MA United States; 3 Department of Medicine Massachusetts General Hospital Boston, MA United States; 4 Department of Women's and Children's Health Uppsala University Uppsala Sweden

**Keywords:** open notes, patient-centered documentation skills, medical student education, 21st Century Cures Act

## Abstract

**Background:**

Patients in the United States have recently gained federally mandated, free, and ready electronic access to clinicians’ computerized notes in their medical records (“open notes”). This change from longstanding practice can benefit patients in clinically important ways, but studies show some patients feel judged or stigmatized by words or phrases embedded in their records. Therefore, it is imperative that clinicians adopt documentation techniques that help both to empower patients and minimize potential harms.

**Objective:**

At a time when open and transparent communication among patients, families, and clinicians can spread more easily throughout medical practice, this inquiry aims to develop informed guidelines for documentation in medical records.

**Methods:**

Through a series of focus groups, preliminary guidelines for documentation language in medical records were developed by health professionals and patients. Using a structured focus group decision guide, we conducted 4 group meetings with different sets of 27 participants: physicians experienced with writing open notes (n=5), patients accustomed to reviewing their notes (n=8), medical student educators (n=7), and resident physicians (n=7). To generate themes, we used an iterative coding process. First-order codes were grouped into second-order themes based on the commonality of meanings.

**Results:**

The participants identified 10 important guidelines as a preliminary framework for developing notes sensitive to patients’ needs.

**Conclusions:**

The process identified 10 discrete themes that can help clinicians use and spread patient-centered documentation.

## Introduction

Reflecting long-standing tradition, medical record notes documenting clinical encounters have primarily served the doctors or other health professionals preparing them. Their diverse functions include accurate documentation of a patient’s unique circumstance, refreshing clinicians’ memories, documenting diagnostic reasoning, communicating cogently with colleagues, justifying charges for encounters, and serving as material for assaying quality of care.

Until about 15 years ago, clinicians prepared such notes rarely with patients or their families envisioned as potential recipients [1]. Since the turn of this century, however, the movement toward more open and transparent communication with patients has grown, and since April 2021, federal rules in the United States now mandate that all patients (with very limited permitted exceptions) are offered online and rapid access to their clinical records, including the notes written by clinicians (“open notes”) [2,3].

Whether one note can serve diverse recipients remains an open question, however, studies suggest room for optimism. A large majority of clinicians experienced with open notes favor their continuation, and few report “dumbing down” what they write [4]. Moreover, extensive survey and qualitative research demonstrate that patients who review their records and read their notes feel more involved in and knowledgeable about their care, report being better prepared for visits, and indicate they are more likely to follow their clinicians’ advice [5-11]. However, words matter, and studies also show that patients can feel judged, stigmatized, or offended by their notes, with potentially adverse effects on the clinician-patient relationship [12-15]. For example, a recent study at 3 diverse health systems found that 1 in 10 patients reported feeling judged or offended by an outpatient note, reflecting the perception that the note contained errors, surprises, inappropriate labeling, or evidence of disrespect [16].

To date, little empirically informed counsel about best practices with respect to patient-centered documentation has been published [17]. Although studies have demonstrated that certain language in notes influences clinician attitudes toward patients and that specific words used can negatively impact the clinician-patient relationship, what language providers should use or avoid has not been clearly described [11,15]. Within the current medical literature, some recommendations have been offered on how clinicians might better prepare for the era of open notes [18]. However, there are no evidence-informed guidelines describing concrete approaches to patient-centered language in clinical notes, and such guidance may be important for developing mindful practices.

In preparation for an educational intervention with medical students and practitioners supervising their work [19], we aimed to address this gap in knowledge and practice by drawing on the perspectives of patients, physicians facile with open notes, medical educators responsible for teaching clinical skills to early learners, and medical residents, who often teach students directly. Following dedicated discussions with these 4 groups and subsequent thematic analysis of their perspectives, we developed a set of guidelines as a preliminary framework for future initiatives aimed at teaching patient-centered documentation skills to medical students, their preceptors, and a broad range of clinicians.

## Methods

We conducted 4 focus groups with discrete groups of individuals to gain an understanding of their perspectives and experiences with written medical documentation. Our goal was to develop guiding principles for best practices in patient-centered documentation skills.

### Design

Our focus groups addressed experiences with written medical documentation (patient notes), with particular attention to the language used in notes. We sought a range of perspectives and structured the groups based on the type of participant. Within their respective groups, the aim was to create opportunities for interaction and comparison of responses among participants [20,21]. This methodology can generate a large volume of responses, and we anticipated a robust discussion of experiences among participants. Although there is no consensus about the ideal number of participants in focus groups, our goal was to recruit between 6 and 10 participants per group [22]. All study procedures received ethical approval in March 2022 and met exempt status both from the Beth Israel Deaconess Medical Center institutional review board (reference number 2022P000079) and the Mass General Brigham institutional review board (reference number 2022P000635).

### Recruitment and Participants

We convened four groups of participants: (1) patients familiar with open notes, (2) practicing physicians facile with open notes, (3) medical student educators involved with teaching clinical skills, and (4) resident physicians working closely with medical students. Between March and April 2022, we used various forms of outreach to recruit participants: For the patient focus group, LS identified, recruited, and contacted patients through email with a flyer attachment. For the group of practicing physicians, TD identified and AG recruited individuals having active experiences with open notes at academic institutions across the country. For the group of medical student teachers, AV and KJ recruited physician educators through the national medical educator list, which included Clerkship Directors in Internal Medicine and Directors of Clinical Skills. For the focus group of residents, AV and KJ identified and contacted through email residents from Beth Israel Deaconess Medical Center and Massachusetts General Hospital who had expressed interest in medical student education. For their time, patient participants were offered a US $100 honorarium, and physicians a US $50 honorarium.

Between April and May 2022, we convened four 90-minute focus groups, each composed of specific types of participants as described above. Focus groups were conducted through Zoom (Zoom Video Communications) and recorded for transcription.

### Format of Focus Groups

Each group followed the same script, with the meetings facilitated by 2 of the study leaders (AV and KJ). Drawing on a variety of stakeholder perspectives, the research team collectively devised the structured interview script. The team included medical educators, practicing physicians, a health services researcher, a patient advocacy researcher, a health services researcher, and a medical ethicist. The script was developed based on the group’s experiential, ethical, and practical experience. To assess face validity, the questions were further honed, refined, and pretested with 4 doctors and 3 patients, leading to further refinements in the wording of questions and prompts (Textbox 1). Groups opened with a standard anonymity disclosure, along with a description of the reason behind the inquiries. We offered participants the opportunity to opt out at any time during the process. Participants were encouraged to set their screen name, however, they felt comfortable and to keep their video off if so desired. To preserve anonymity, we asked participants not to mention each other’s names during the session.

**Focus group questions for patient and physician groups.**

(10 minutes per question)

**Question 1: What do you recall from any previous learning experiences that focused on writing notes that patients will read, or reading notes from the perspective of a patient?**

- For the patient group: “What do you recall from your experience of reading your clinical notes?”
- Follow-up question: What are some concrete examples of this?

**Question 2: What should early medical students know about writing notes that will be useful to patients and encourage partnering or engagement in their care?**

- Follow-up question: What are some concrete examples of this?

**Question 3: What should early medical students know about writing notes with words or phrases that could be harmful to patients or their relationship with their physician?**

- Follow-up question: What are some concrete examples of this?

**Question 4: What should early medical students know regarding how medical vernacular or acronyms may be perceived by patients?**

- For patient group: “What has been your experience when reading notes containing medical vernacular or acronyms?”
- Follow-up questions:
- What are examples of vernacular that should be avoided due to possible patient harm or creating unwanted bias?
- Are there common acronyms that should be avoided?

**Question 5: What should early medical students know about including the patient’s voice in the notes?**

- For patient group: Describe some ways in which a physician could write your words, or ensure that the writing of your medical concerns represents your lived experience.
- Follow-up question:
- What documentation approaches could a student follow to help the patient feel authentically seen and heard when reading their clinical notes?

**Question 6: What should early medical students know about how words or phrases in clinical notes may convey bias?**

- For patient group: “What has been your experience in reading notes with words or phrases that you feel convey bias?”
- Follow-up question: What are some concrete examples of this?

**Question 7: What key content areas are more sensitive for patient readers and should require students to receive specific guidance on documentation?**

- Follow-up question:
- What topics or themes should receive special attention and teaching?
- Are there any key areas that should be omitted or avoided in notes unless discussed and reviewed with faculty?
- Prompt examples: race, obesity, firearm ownership, gender identity and health, sexual identity and health, substance use, and so on.

(5 minutes for wrap-up and debrief and closing thoughts)

### Analysis

We designed and conducted an inductive thematic content analysis of the transcribed focus groups [23]. This approach was used because it is particularly appropriate for analyzing textual data by identifying patterns, themes, or categories that emerge from the data itself, rather than imposing predetermined categories or codes. This approach facilitates the identification of nuanced themes arising directly from the data, making it particularly useful when exploring new or less understood phenomena [24]. Responses were analyzed by 2 members of the research team (AV and KJ). Both are medical educators and general internists in the United States with experience in sharing online access to patients’ health records (AV is an inpatient physician and KJ is a primary care physician). The research team was diverse in age and background: the lead author and one of the coders (AV) identified as of Indian background, another author (AG) identified as having Cuban descent, and the patient-researcher author (LS) identified as having both physical and cognitive disabilities.

First, AV and KJ read the transcripts to familiarize themselves with the responses. Second, AV and KJ independently created codes through the selection of excerpts from each of the 4 focus groups. The codes were then reviewed jointly by AV and KJ to come to a consensus. Using the common list, new excerpts from each of the 4 transcripts were coded by AV and KJ, and the codes were further refined until a consensus was reached. Subsequently, first-order codes were grouped into second-order themes based on commonality of meaning. Representative comments for each theme were identified by authors AV and AG.

### Ethical Considerations

All study procedures received ethical approval in March 2022 and met exempt status both from the Beth Israel Deaconess Medical Center institutional review board (reference number 2022P000079) and the Mass General Brigham institutional review board (reference number 2022P000635). This study did not meet criteria for human subjects research at either institution and thereby was deemed exempt.

## Results

### Overview

A total of 27 individuals participated in the focus groups: 8 patients (5 women, 2 men, and 1 individual identifying as gender nonbinary), 5 physicians with experience preparing open notes (2 women and 3 men), 7 medical student educators (6 women and 1 man), and 7 resident physicians (6 women and 1 man). The participants from the patient, physician, and medical educator groups were from diverse geographical regions of the United States. Based at large academic centers, physician participants represented different clinical specialties including internal medicine, pediatrics, and behavioral health. Educator participants were all involved with either leadership or teaching foundational clinical skills at various medical schools in the country. The resident physician participants were recruited from 2 large academic health centers in the greater Boston area.

Using the iterative coding process [25], we identified 10 major themes that could serve as guidelines for patient-centered documentation (Textbox 2). We discuss these in greater detail below, with representative comments illustrative for each theme in Table 1.

**Checklist of guidelines for patient-centered documentation: major themes.**

**Themes**

- Use person-first language.
- Refer to your patients as how they want to be identified.
- Avoid abbreviations and acronyms, especially if not officially approved by the practice.
- Say what you write and write what you say.
- Verify past history information before recording it in the note.
- Avoid words that may convey bias or judgment.
- Keep descriptions of physical examinations objective.
- Empower your patients with encouraging words and clear next steps.
- Pay close attention to sensitive topics, including but not limited to sexual history, trauma history, substance use history, mental health, or illness.
- Write from your perspective.

**Table 1:** Examples and suggestions for each identified best practice for patient-centered documentation.

Theme	Examples and suggestions
Use person-first language	I don’t see necessarily a boundary between the language you use to describe a patient out loud and what you write in your note. It’s all part of a single way that you frame patients. It’s not a diabetic patient. It’s a patient with diabetes. I really don’t think about the way that I document my notes differently than the way that I just think about my patients, which is to think about them in a person‑centered way. [#2, Medical Educator] Even though I’ve lost weight, the word morbidly obese is in every note, every note I see. That doesn’t bring me joy or comfort me or get me to want to interact in a positive manner. My issues have nothing to do with my weight and never did. I developed a MRSA staph infection; it was a healthcare-acquired infection.There are certain things where weight doesn’t always factor in, and you don't always need weight. [#1, Patient]
Refer to your patients as how they want to be identified	I have found the doctors that I have currently are really good about using he/him pronouns for me and referring to me as using masculine identifying language. I have had providers who are not. Never knowing what I’m going to run into in those notes is very anxiety-inducing on the gender part alone, right? Let alone, does this person think that my gender is influenced by or influencing any of my other health issues? [#5, Patient] I tend to write and call the patient whatever they like to go by, so I’ll say like, Bill is a 75‑year‑old man, instead of Mr. Smith, for instance. For nonbinary or transgender patients, I try to ask them what gender they would like me to document rather than just assuming it’s transgender male. Then in the social history and medical history, perhaps elaborating on it more, but trying to give them voice in what I’m documenting. I also think that mentioning race without having any clear connection to anything should be discouraged completely. [#1, Resident]
Avoid abbreviations and acronyms, especially if not officially approved by the practice	I was struck by the idea that it almost felt, as a medical student, as if you were being inducted in a secret society. It was this secret language that you now all understood, and that’s what unified everyone who was part of that. It really is just so unnecessary at this point. I think what’s already come out here is that there clearly are regional differences. I’ve never heard of “MOP” [“mother of patient”], but “ISO”[“in the setting of”] is rampant around here, so clearly, there are differences in terms of some of the abbreviations that are used in specific regions and areas, and we can’t then even understand each other’s language. [#2, Physician] Just the other day, we were talking about PSA and how it’s prostate‑specific antigen, but also pseudomonas, and also pseudoaneurysm. If a patient reads PSA and looks that up for themselves on Google, they’re going to find a million things that could be. If you’re going to use an acronym, maybe don’t use ones that have multiple different meanings even in the medical world, and the most important parts of their diagnoses should not be abbreviated. [#1, Resident] Medical jargon and all this evolved as a way of having more succinct communication, being able to communicate among clinical teams. I think there is some value to that, but I think what’s important for a lot of educators to keep in mind is, this is an area where the students are actually—while they’re learning, they also should, in some ways, be teachers for all of us who have been doing this for some time, right, and how they can take their lack of being indoctrinated by the system and bring that from the bottom up. [#3, Physician] Then some acronyms. Point out we say SOB for shortness of breath, and, obviously, has other meanings. [#2, Resident]
Say what you write and write what you say	There probably shouldn’t be anything in the note that hasn’t been discussed. If you’re telling someone a diagnosis, I don’t think you should go back in the note and say, “And this diagnosis is terminal” if you haven’t discussed it with the patient. Knowing that the patient is likely going to go back and read the note, I don’t think that it should be put in the note. Or, if the physician feels like it’s important that it’s put in the note, make sure it’s discussed with the patient as well. There’s no point in writing a good note if none of it was even said to the patient. [#7, Patient] When we have a discussion and he puts it right in my note right then and there, those are kinds of things that help me. Today I had a call from the pharmacist about a med. They would have given me the wrong med if I wouldn’t have stood up for myself and wouldn’t have known what I was supposed to have, and wouldn’t have had my notes there, because basically I read it right from my note to him. I think the notes become more and more valuable. [#1, Patient]
Verify past history information before recording it in the note	We’ve evolved as people from year 1 to year 5 over the course of a relationship of knowing a doctor, and our social histories often don’t reflect that change. What could have been pertinent 5 years ago for a patient might not be anymore, and depending on what they were experiencing at that point in time, could be biasing. [#1, Resident] So now it’s on my to-do list when I go to appointments to make sure that’s changed. Really, that shouldn’t be a priority of mine when we go into medical appointments to say, “Hey, I read the note, and this needs to be changed, ‘cause this doesn’t represent who I am.’ I think everyone has assumed the person before them has done it, and it’s accurate. It takes a lot of time and a lot of effort and a lot of energy for us to be able to correct mistakes that are in our patient portals, our notes. I think it’s 1 of those things where I just want to remind medical students, you can do 2 things to check with patients. Like, “Hey, I have all this down. Is that right?” Two, “If you see anything in here that’s wrong, send me a MyChart message or EHR or whatever message, so I can fix it. [#5, Patient] Some things that I notice that get copy‑forwarded a lot in notes that create bias in the reader, 1 would be substance use disorders, and not really specifying or clarifying it, right? It will just say, “Polysubstance use disorder.” Things like that, I think definitely have the ability to bias. [#1, Resident]
Avoid words that may convey bias or judgment	You don’t need to use the word complain. It’s a pretty negative connotation. Patient “reports”, or just “is having”. I think avoiding the word complaint is pretty important.” [#2, Resident] I think, if you’re ever thinking about using a term like that, it should be more about, what are the barriers this patient has to achieving care, and talking more about that and not just using 1 word. Using more language is actually helpful, and med students often have more time to actually ask patients about these things and have a conversation. I think that could be a really empowering point for medical students to learn more about the determinants of health that are affecting the patient more so than being like, “This patient doesn’t take their medications. [#5, Resident] I also think that mentioning race without having any clear connection to anything is—should be discouraged completely. It’s such a part of vernacular, especially more physicians who are trained in a different era, so we’ve been actively encouraging students not to include that unless it has a very clear reason. [#2, Medical Educator] I could imagine, sometimes, quotation marks are used to just directly capture what the patient said. I think it’s about striking that balance of bringing the patient language in and having some of it as verbatim as directly as possible so that the patient can see what they said was actually recorded, but then a key part of what we’re doing in notes is really clinical translation. It’s about going from verbatim conversation to clinical processing and then recording our clinically processed thoughts. [#3, Physician]
Keep physical examination descriptions objective	General appearance is very tricky, and I think we all have to ask ourselves, when is that relevant and why, and then really distill it down to what’s clinically meaningful in the least judgmental way possible. Is it important that the patient is disheveled or older than stated age or has poor hygiene? It might be, but then you have to figure out, how do you relay that in the most respectful manner possible? [#5, Medical Educator]
Empower your patients with encouraging words and clear next steps	There’s a book called “The 15 Minute Hour” that’s geared toward primary care providers, and 1 of my favorite takeaways from it that has always stuck with me over the years is the idea of using the word “yet” at the end of a sentence or a phrase. It’s supposed to emphasize that you’re not at a dead end. You can still do this. The patient has not been able to quit smoking yet. The patient has not lost weight yet. [#5, Physician] I somehow think that I would like medical notes to be imbued with a different perspective, that we’re trying to teach patients to be friends with their disease…[…]…disease is not the enemy. [#4, Patient] I have heard from some patients that we had engaged in providing feedback on our student notes that they would really like, in reading the assessment and plan, to have some understanding of what they can do next and what the evaluation, diagnostic evaluation plan means for them. Maybe some additional content around health coaching and steps that they can take in their lives to support their health too. It could be a great way to extend that partnership beyond the visit. [#5, Medical Educator]
Pay close attention to sensitive topics, including but not limited to sexual history, trauma history, substance history, mental health or illness	Some advice I’ve gotten from the psychiatry consulting team at my primary care practice is […] to just state the type of trauma it was, the years that it happened, the relation to the abuser, and what the patient went through….[…]…There’s no need to go into extreme detail and quotations about what the patient confides with you in clinic about, that you can still have a therapeutic bond…[…]…instead aim to strike a balance of what’s useful for other providers and not needed for the patient to be reading about themselves. [#7, Resident] There are certainly very specific topics around mental health, around sexually transmitted illnesses, reproductive health, substance use, et cetera, that definitely should be addressed in a particular manner that respects the patient’s privacy… […]…early learners could certainly benefit from understanding what the implications of access to that information could be. [#2, Physician] […] documenting your full differential diagnosis, where you might have a patient who is coming in for what seems like a respiratory illness or pneumonia, but maybe under differential, you also have a rare lung disorder or lung cancer. It’s trying to strike that balance…[…]…trainees, as part of their training, are taught to document the full differential …[…]…this is an area where we’re all still trying to figure out the best practices because you do want to indicate to some degree that you are thinking about these other diagnoses, but in the real world […]you might not start with an initial visit by sharing that this may be cancer, but it may be something you bring up on a follow-up visit when your initial working diagnosis doesn’t seem to be correct and it seems more serious than that. I know the official recommendation has been to just document what you discuss…[…]…I think it’s more complicated than that. [#3, Physician]
Write from your perspective	Something I’ve been experimenting with a lot is using the first person in the assessment and plan, which I don’t know if I was ever explicitly told not to in medical school, but I feel like I thought I wasn’t supposed to. Over the past year, I’ve started to come around to this idea of, the objective is the objective, but the assessment and plan is my medical opinion. A lot of times, I’ll say, “I'm worried about”, or, “I think this might be at play. I think it’s unlikely, but maybe cancer. I think anxiety might be driving some of this.” I feel like that really couches it as, this is my opinion of what’s going on. It doesn’t negate what the patient thinks. [#5, Physician]

### Use Person-First Language

Across all 4 focus groups, participants agreed that patients should be referred to as individuals with certain clinical conditions, rather than identifying them primarily by their medical condition (Table 1). Some patients identified a preference for the use of discrete numbers when addressing a person’s weight, rather than describing the individual as “obese” or even “with obesity,” and to be mindful of whether weight needs to be in the note if not relevant to issues being addressed (Table 1). All participants from the patient group described the importance of being mindful of phrases and words in notes that can trigger trauma for patients. Finally, participants in all groups identified descriptions of words and phrases in the medical record considered potentially depersonalizing or dehumanizing.

### Refer to Your Patients as How They Want to Be Identified

The participants recommended that patients be identified according to their own expressed preferences. Participants from the resident physicians’ group suggested that when first introducing oneself to a patient, one should request and document how the patient wishes to be identified in the record. Patient participants noted that honorifics and gender identity should never be presumed (Table 1). In addition, patient participants explained that patients may want to be identified by their life roles, such as their profession, or background, in addition to honorifics and names. Medical educators recommended that introductory “History of Present Illness” sentences should mention factors important to understanding and planning the care of that person at that point in time, with the understanding that these factors are dynamic and change over time. Physicians familiar with open notes suggested it is important to be thoughtful and deliberate about whether to include demographic factors, epidemiologic factors, and medical and social history in the opening sentences of a note, given the effect such information may have on framing how a patient, family member, or other clinician reads the note.

### Avoid Abbreviations and Acronyms, Especially if not Officially Approved by the Practice

Participants in all groups felt that medical shorthand can cause confusion and misinterpretation by patients. Physician, resident, and educator participants noted that while some abbreviations are widely understood within health care, others are interpreted differently within and across a given specialty. They suggested that this, in turn, could lead to clinician confusion, thereby further amplifying the risk of patient confusion (Table 1). Physician participants noted that learning to use medical shorthand in notes was akin to being inducted into a “secret society” (Table 1). Illustrative examples suggested by the 4 focus groups included: “SOB” (shortness of breath), “F/U” (follow-up), “ISO” (in the setting of), “NAEON” (no acute events overnight), and “MOP” (mother of the patient). In addition, physician participants pointed out that shorthand for certain medical diagnoses was problematic, given the frequent lack of clarity. They cited “HFrEF” (heart failure with reduced ejection fraction); “HFpEF” (heart failure with preserved ejection fraction); and “AKI” (acute kidney injury). Several residents discussed the risk that abbreviations and acronyms may perpetuate discriminatory biases within notes. Medical educator participants noted that this was an area in which practicing clinicians can learn from students, given that many have not yet been inducted into contemporary systems of medical vernacular. Finally, the medical educators noted that even if abbreviations were minimized, notes still may not be completely understandable, underscoring the need for open communication with patients.

### Say What You Write and Write What You Say

Patients universally stressed the importance of notes accurately reflecting what was done and discussed during a visit. Residents and physicians emphasized that clinicians should be mindful about not documenting issues, such as possible diagnostics considerations, that were not discussed directly with the patient, and that this principle should be used consistently for all documentation (Table 1). Medical educators identified the importance of guiding learners carefully in interactions between documenting explicit clinical reasoning with robust documentation of differential diagnoses and setting expectations for patients. They suggested that clinicians point out to their patients that some notes are intended to be comprehensive and may include diagnostic possibilities that are unlikely, but nevertheless important to record. In contrast, patients suggested there are situations in which clinicians should not write too much about a potentially sensitive topic that may trigger a harmful response in patients. Instead, patients proposed clinicians might record that a discussion of a “challenging topic” took place during the visit; although details might be excluded, such reference would remind the patient of the conversation. On the other hand, several patient participants noted that accurate and detailed representations of a visit may help empower patients with managing their care, strengthen their agency, and enhance their understanding of medical recommendations.

### Verify Past History Information Before Recording it in the Note

Another theme was the importance of verifying patients’ past information in the note to avoid “note bloat” (ie, copy and paste from previous notes). Participants from all groups identified “cutting and pasting” as increasing the risk of mistakes. Notably, all patients agreed that the “note bloat” phenomenon can have a negative impact on how patients view their notes, and possibly on their relationship with clinicians, resulting in their feeling the need to advocate for themselves to ensure accurate representation of their stories (Table 1). Several patient participants suggested that copying over all aspects of the social history when not relevant to a specific visit might trigger trauma since this section of the note can replicate and reiterate sensitive information. Two residents noted the issue of “copy-forward” (copying previous information from the record into new notes), thereby propagating bias and stigma, and potentially risking mistrust of the clinician. Medical educators noted that untruthful documentation, at times propagated by copy-and-paste templates, can lead to mistrust in the patient-physician relationship and adversely impact care.

### Avoid Words That May Convey Bias or Judgment

With striking overlap across all 4 groups, participants offered many examples of words and phrases that may reflect bias or unfair judgment of patients. Suggestions about common phrases and words to avoid included clinical vernacular such as “complains,” “denies,” “claims,” “refuses,” and “endorses.” Participants recommended neutral and judgment-free words such as “says,” “reports,” “does not report,” “concerns,” and “did not tolerate.” Several medical educators suggested renaming “chief complaint” as “chief concern.” Participants also discussed problems with labels, such as “poor historian,” “noncompliant,” “difficult patient,” “nonadherent,” “uncooperative,” “having poor health literacy,” and “left AMA.” Patient participants agreed on the importance of describing explanations for observed behaviors, rather than using judgmental language, such as, “Mr. X is unable to take his insulin most days of the week due to inability to refrigerate the medication at his place of work.” Consideration about how to document race was another common concern. Given that race is without clear relevance to most medical concerns, many believed it should not be included in the written documentation. Finally, the pros and cons of using quotation marks and the patients’ own language were discussed. Participants noted that doing so would accurately capture what was said. However, in certain contexts, this might be interpreted as sarcastic, questioning, or making light of what the patient communicated, and participants recommended that quotation marks should be used carefully.

### Keep Descriptions of Physical Examinations Objective

Another emergent theme was ensuring that descriptions of physical examinations avoided phrases that could be perceived as offensive. Examples included: “disheveled,” “older than stated age,” “obese”; and judgmental descriptors, such as “argumentative.” Participants recommended omitting descriptors such as “pleasant” or “delightful,” in part because the absence of such terminology might inadvertently suggest to the reader that the patient is “unpleasant.” Several participants urged considering whether language pertaining to appearance is helpful. All recommended avoiding the words “obese” or “morbidly obese” and to first consider whether the information is relevant to an issue at hand. If so, they recommended quantitative descriptors, for example, BMI or the actual weight.

### Empower Patients With Encouraging Words and Clear Next Steps

Participants suggested patients often benefit from reading encouraging words and the next steps regarding their conditions. Physicians noted that empowering language, particularly in the “Assessment and Plan” section of a note, could stimulate patients to engage in their care more actively. One physician suggested adding the word “yet” at the end of the sentence or phrase, signifying progress with a specific goal (Table 1). Patients agreed that empowering notes could facilitate partnership with the medical team. Medical educators suggested adapting notes to ensure they capture expert or emerging clinical reasoning, while still engaging patients in the next steps of a plan. Relatedly, several participants suggested using notes to embed reminders to patients, their care partners, and other members of their care team by stating explicitly what was recommended during the visit, especially with respect to specific next steps and goals.

### Pay Close Attention to Sensitive Topics, Including but not Limited to Sexual History, Trauma History, Substance Use History, Mental Health, or Illness

Another emergent theme was mindfulness in documenting topics such as substance use history, mental health and illness, gender identity, sexual identity and health, history of trauma, disagreements between patients and clinicians, significant illnesses, and comprehensive diagnostic reasoning. Medical educators noted the challenges of teaching early learners how best to document such information in the social history section. Physicians noted the importance of documenting any disagreements with patients in an objective and respectful manner.

### Write From Your Perspective

Medical notes, in particular the “Assessment and Plan” portion of the note, reflect the perspective of the health care professional at a given point in time. Participants uniformly noted that the patient’s perspective should also be included, and that descriptions should be factual and based on direct observations. Physician and resident participants alike suggested the use of the first-person pronoun “I” when writing the “Assessment and Plan,” as well as using the phrase “at this point in time.” Physicians advised that this wording signals that diagnostic considerations may change as a situation evolves.

## Discussion

### Principal Results

At a time of increasingly open and transparent communication between patients and clinicians, a growing body of research demonstrates the importance of words and phrases used in clinical notes, and the risk of bias and offense to patients. Patients who read their notes feel more involved in their care, better prepared for visits, and are more likely to follow their clinician’s recommendations [4-10]. Just as importantly, patients can experience negative effects from their notes due to language perceived as judgmental, offensive, or stigmatizing [11-14]. Language in notes can also negatively influence other clinicians reading the note, leading to bias and impact on the patient’s care [11]. To our knowledge, this is the first study attempting to define concrete guiding principles on best practices in patient-centered documentation. Informed by a qualitative analysis of active and interactive discussion among patients and clinicians, this inquiry, as described in detail above and in Textbox 2 and Table 1, identified 10 discrete guidelines for patient-centered documentation.

This inquiry was stimulated by our interest in facilitating open and transparent communication among health professionals and patients from the very beginning of a clinician’s career. Medical school curricula and residency programs are just beginning to introduce the concept of patient-centered documentation, with few providing specific guidance [26-28]. Concrete advice is especially important for early medical learners. Moreover, many faculty and residents who teach medical students are also relatively new to the practice of open notes. Developing and describing guidelines, accompanied by clear and detailed examples, can help faculty both learn what we hope will evolve as best practices and teach students these skills more easily, creating an opportunity for standardized assessment of notes based on a checklist reflecting the guidelines. In addition to providing direct teaching and feedback, the presence of concrete guiding principles will allow faculty to role model these skills in patient interactions for their learners. Furthermore, given the importance and impact of language in documentation, we believe the guidelines identified in this research will serve all clinicians well.

The checklist this study generated has already been implemented within the first-year foundational clinical skills course students undertake at our medical school [19]. Students are introduced to the background and value of open notes, followed by the checklist of guidelines. Over the timeframe of this course and beyond into their core clinical training, students are expected to write patient notes using these guides and are subsequently assessed on the quality of their notes through a rubric based on the checklist. Faculty preceptors in this first-year course are also encouraged to use this checklist, with the aim of reviewing student notes and providing feedback based on these 10 themes.

### Limitations

Our study has several limitations. First, owing to time constraints on the duration of the study caused in part by the COVID-19 pandemic, we did not conduct focus groups until data saturation was reached. Second, due to the need to develop this preliminary framework before the delivery of a patient-centered documentation curriculum for students, the data analysis was restricted to 2 reviewers only, limiting the validation of the data. Third, the small number of participants likely influenced the results. Fourth, and relatedly, a fuller spectrum of diversity including geography, clinical background, and health background, could produce more varied views and depth of experience with open notes. Fifth, studies have demonstrated that fewer patients with low income, limited education, or poor English proficiency are active users of the electronic health record, limiting our participants’ understanding of the note-reading experience of these populations [29-31]. Our preliminary guidelines may therefore be limited when it comes to generalizing to wider patient populations, and future guidelines should seek to address this concern. Finally, the development of new charting practices and auditing may come with considerable resource demands. Typically, guidelines that assess resource-intensive interventions are more likely to include an assessment of implementation costs, real and intangible. It would also be useful to discuss the potential risks of open notes and any context-specific adjustments for special populations or clinics. This is, however, beyond the scope of this study.

### Conclusions

The guidelines identified are preliminary. As our understanding grows, we expect clinicians will learn how to write notes in ways that patients find increasingly useful. In addition, there are nuanced areas to consider in various specialties and different patient populations that may require further adaptation of these guidelines. To this end, clinicians and patient advocates partnering to co-design medical education will be important, as it was in this study. Ideally, in the future, we will learn to teach practitioners effective documentation through a common language and shared set of standard expectations. Although we expect norms and practice techniques to evolve, our study presents an initial attempt to develop practical and respectful guidelines for patient-centered documentation.

## Abbreviations

AKI: acute kidney injury
HFpEF: heart failure with preserved ejection fraction
HFrEF: heart failure with reduced ejection fraction
